# Morphological correlates to cognitive dysfunction in schizophrenia as studied with Bayesian regression

**DOI:** 10.1186/1471-244X-6-31

**Published:** 2006-08-10

**Authors:** Glenn Lawyer, Håkan Nyman, Ingrid Agartz, Stefan Arnborg, Erik G Jönsson, Göran C Sedvall, Håkan Hall

**Affiliations:** 1 Karolinska Institutet, Department of Clinical Neuroscience, Karolinska Hospital, Stockholm, Sweden; 2 Department of Psychiatry, Oslo University, Oslo, Norway; 3 Department of Numerical Analysis and Computer Science, Royal Institute of Technology, Stockholm, Sweden; 4 Uppsala Imanet AB, Uppsala, Sweden

## Abstract

**Background:**

Relationships between cognitive deficits and brain morphological changes observed in schizophrenia are alternately explained by less gray matter in the brain cerebral cortex, by alterations in neural circuitry involving the basal ganglia, and by alteration in cerebellar structures and related neural circuitry. This work explored a model encompassing all of these possibilities to identify the strongest morphological relationships to cognitive skill in schizophrenia.

**Methods:**

Seventy-one patients with schizophrenia and sixty-five healthy control subjects were characterized by neuropsychological tests covering six functional domains. Measures of sixteen brain morphological structures were taken using semi-automatic and fully manual tracing of MRI images, with the full set of measures completed on thirty of the patients and twenty controls. Group differences were calculated. A Bayesian decision-theoretic method identified those morphological features, which best explained neuropsychological test scores in the context of a multivariate response linear model with interactions.

**Results:**

Patients performed significantly worse on all neuropsychological tests except some regarding executive function. The most prominent morphological observations were enlarged ventricles, reduced posterior superior vermis gray matter volumes, and increased putamen gray matter volumes in the patients.

The Bayesian method associated putamen volumes with verbal learning, vigilance, and (to a lesser extent) executive function, while caudate volumes were associated with working memory. Vermis regions were associated with vigilance, executive function, and, less strongly, visuo-motor speed. Ventricular volume was strongly associated with visuo-motor speed, vocabulary, and executive function. Those neuropsychological tests, which were strongly associated to ventricular volume, showed only weak association to diagnosis, possibly because ventricular volume was regarded a proxy for diagnosis. Diagnosis was strongly associated with the other neuropsychological tests, implying that the morphological associations for these tasks reflected morphological effects and not merely group volumetric differences. Interaction effects were rarely associated, indicating that volumetric relationships to neuropsychological performance were similar for both patients and controls.

**Conclusion:**

The association of subcortical and cerebellar structures to verbal learning, vigilance, and working memory supports the importance of neural connectivity to these functions. The finding that a morphological indicator of diagnosis (ventricular volume) provided more explanatory power than diagnosis itself for visuo-motor speed, vocabulary, and executive function suggests that volumetric abnormalities in the disease are more important for cognition than non-morphological features.

## Background

Approximately 1% of the population is diagnosed as having schizophrenia sometime during their lifetime (for details, see [1]). Schizophrenia typically causes great suffering and loss of quality of life for the patients, their families and society at large. The cost for the society is counted in billions of dollars [1]. Most of the patients are chronically ill or have a fluctuating course, and few come to live a relatively normal life emotionally, socially, or occupationally once the disease has developed.

Neuropsychological studies clearly show that patients with schizophrenia suffer impaired cognitive performance. There is some consensus among researchers that the main domains of impairments are in attention, learning, and executive functions [2]. Magnetic Resonance Imaging (MRI) techniques have provided evidence for brain morphological changes in schizophrenia [3-5]. The most prominent changes are the enlarged ventricles, but among other structural differences are volume reductions of the medial temporal lobe and the frontal lobe, and changes in subcortical brain regions including the cerebellum, basal ganglia, corpus callosum, and thalamus.

A number of theoretical frameworks that attempt to understand relationships between neuropsychological deficits and brain morphological changes in people with a diagnosis of schizophrenia have arisen over the years. Cognitive processing is widely regarded to take place primarily in the brain cortical gray matter, and significant deficits in cortical volumes [3,4] and thickness [6] have been identified in schizophrenia. Other evidence points to abnormalities in neural circuitry, leading to the "disease of connectivity" hypothesis [1]. While Andreasen [1] discusses cortical-thalamic interactions and cerebellar functions, disruptions in connectivity could also be associated with abnormalities in the basal ganglia or in deep white-matter tracts such as the corpus callosum. A related branch of research considers cerebellar and specifically vermis deficiency [7-9], which has possible implications for sequencing ability, working memory, and eye movement [10,11].

Each of these just mentioned possibilities is biologically plausible and supported by rigorous measurement and testing. Such testing, however, is generally performed within the specified theoretical framework. We thought it of interest to perform one advanced statistical analysis that encompassed all of these theoretical frameworks and allowed for complex interactions between the morphological features underlying cognition.

This work studies relationships between brain structure volumes and neuropsychological performance while allowing those relationships to differ in schizophrenia. A Bayesian decision-theoretic analysis [12] was used to explore a linear model, which had neuropsychological tests as outcomes and brain morphological measures as covariates. Interaction terms in the model allowed for alterations in the relationship between morphometry and neuropsychological performance in the patients. The analysis identified those morphometric factors and interactions that were statistically relevant to neuropsychological test scores.

## Methods

### Subject characterization

Subjects were unrelated Caucasian individuals living in the north-western part of Stockholm County drawn from a larger, previously described cohort [13]. The subjects were recruited at the Department of Clinical Neuroscience, Karolinska Hospital, Stockholm, Sweden, and investigated between August 1999 and Spring 2003. Subjects were assessed for lifetime psychiatric diagnosis [DSM-III-R or DSM-IV, [14,15]] and geographical origin using reviews of hospital case notes and clinical structured interviews [16,17] performed by psychiatrists trained in Sweden.

There were 71 patients (57 men, 14 women, mean age ± S.D., 40.8 ± 7.6 years) and 65 control subjects (39 men, 26 women, mean age ± S.D., 44.1 ± 7.7 years). The patients fulfilled DSM-III-R or DSM-IV diagnoses of schizophrenia (SCZ, n = 56), schizoaffective disorder (SCA, n = 8), or psychosis not otherwise specified (NOS, n = 7). None of the control subjects fulfilled diagnostic criteria for any psychosis diagnosis or prepsychological disorder. Of the patients 64 received antipsychotic medication at the time of the investigation, whereas seven did not. In the patient group the mean age of onset and duration of illness was 24.3 (± 5.0) and 16.4 (± 8.3) years, respectively.

Subjects were characterized by neuropsychological performance and a number of brain morphological measures, as detailed below. Many of the morphological measures were manually traced structures. The full compliment of morphological measures was only available on a subset of the subject group, which contained 30 patients (SCZ n = 22, SCA n = 4, NOS n = 4) and 20 controls.

The study was conducted in accordance with the Declaration of Helsinki and approved by the Ethics Committee of the Karolinska Hospital and the Swedish Data Inspection Board ("Datainspektionen"). All subjects participated after giving informed written consent.

### Neuropsychology

The Cognitive Performance Indicator (CPI) is a semi-computerized neuropsychological test battery, constructed to give an overview of important cognitive functions in patients with schizophrenia. Tests covering six functional domains were used for this investigation. The Rey Auditory Verbal Learning Test (RAVLT) affords an analysis of learning and retention by a five-trial presentation of a 15-word list (list A), a single presentation of a distractor list (list B), and two postdistractor recall trials – one immediate and one delayed [18]. A classical measure of vigilance is included in the form of a 150-item version of the Continuous Performance Test – Identical pairs (CPT) [19]. Visuo-motor speed was measured by the Trail Making Test Form A (TMTA) and the more demanding version Form B (TMTB), which also includes the requirement to shift between different aspects during performance [18]. The latter version is thus in some respects also measuring the ability to monitor on-going behavior, which may be regarded as an aspect of executive function. Letter-Number-Sequencing (LNS), a subtest from the WAIS-III, adds a very specific measure of working memory capacity [20]. The Vocabulary (WAIS-R) subtest, also part of the WAIS series, is regarded as a "hold test", roughly assessing premorbid functional level [21]. The 64-card version of the Wisconsin Card Sorting Test (WCST) [22] measures aspects of executive functions. A summary of the tests can be found in Table 1.

**Table 1:** Neuropsychological tests.

	**CPI test**	**Explanation**	**References**
	Verbal learning		Lezak, 2004 [17]
1	RAVLTA1	Rey Auditory Verbal Learning Test (five trials using the same 15 words)	
2	RAVLTA2		
3	RAVLTA3		
4	RAVLTA4		
5	RAVLTA5		
6	RAVLTATOT	Sum of A1-A5	
7	RAVLTB	Distractor (new words)	
8	RAVLTA6	Immediate recall (same words as in A1 – A6)	
9	RAVLTA7	Delayed (20 min) recall	
	Attention, vigilance		Cornblatt et al., 1989 [18]
10	CPT	Continuous Performance Test – Identical Pairs	
	Visuo-motor speed		Lezak, 2004 [17]
11	TMTA	Trail Making Test, form A	
12	TMTB	Trail Making Test, form B	
	Working memory		Wechsler, 1997 [19]
13	LNS	Letter-Number-Sequencing	
	Functional level		Wechsler, 1981 [20]
14	Vocabulary, WAIS-R	Subtest from Wechsler Adult Intelligence Scale	
	Executive functions		Heaton et al., 1993 [21]
15	WCST CAT	Wisconsin Card Sorting Test (64 card version), completed categories	
16	WCST total err	Number of errors	
17	WCST pers err	Perseverative errors	
18	WCST pers resp	Perseverative responses	

The Cognitive Performance Index (CPI) is a battery of standardized neuropsychological tests. The above-listed tests, covering six functional domains, were selected from the full CPI to give an overview of cognitive abilities in our subject group.

The tests were administrated in a standardized order. The psychologists who administered the tests were trained and supervised not to induce undue stress or fatigue in the patients, thus securing optimal performances. Results were immediately entered into in a database.

### Brain morphology

The brain of each individual was characterized with respect to volume of anatomical regions, using magnetic resonance imaging (MRI) and computerized image analysis. A standard protocol was established to be used in conjunction with the tissue classification (segmentation) program BRAINS [23-25]. The subjects were investigated in a 1.5 Tesla GE signa Echo-speed (Milwaukee, Wis., USA) system at the MR Research Center, Karolinska Hospital, Stockholm, Sweden [26,27]. T1-weighted images, using a three dimensional spoiled gradient recalled (SPGR) pulse sequence, were acquired with following parameters: 1.5 mm coronal slices, no gap, 35° flip angle, repetition time (TR) = 24 ms, echo time (TE) = 6.0 ms, number of excitations (NEX) = 2, field of view (FOV) = 24 cm, acquisition matrix = 256 × 192. T2-weighted images were acquired with the following parameters; 2.0 mm coronal slices, no gap, TR = 6000 ms, TE = 84 ms, NEX = 2, FOV = 24 cm, acquisition matrix = 256 × 192. Selected pulse sequences were used in one scanning session lasting approximately 50 minutes. All scans were evaluated for gross pathological changes by a neuroradiologist.

Regional measurements were ascertained by transformation of MRI data into Talairach space [25,28]. The Talairach boxes were assigned to specific regions corresponding to the frontal, occipital, parietal, temporal, and occipital lobes, and to the subcortical region. The segmented tissue class volumes of each Talairach box, the cerebral-spinal fluid volume of the ventricles I, II, and III, and the intracranial volume were measured automatically and separately [24,29]. A number of regions (corpus callosum, caudate, putamen, hippocampus, cerebellum, the posterior superior, posterior inferior, and anterior vermis, and cerebellar tonsil) were manually delineated on a randomly selected subset of the sample. The quantitative analysis was performed blinded with regard to the two diagnostic categories.

Note that all of the measurements depended to a certain degree on the operator. Representative voxels for venous blood, the internal capsule, and thalamus were manually selected. The delineation used to determine the intracranial volume was the product of systematic and careful editing of an automated skull stripping procedure. The borders of the talairach boxes were manually placed.

Reproducibility and reliability of this procedure have been ascertained previously for both automatic segmentation and manual tracing [8,27,29]. Briefly, two independent operators, specialists in psychiatry with at least one year of postdoctoral training, and blinded to subject diagnosis and identity, performed the automatic segmentation as well as the manual tracing. The intra-class correlation coefficient (ICC) from ten scans investigated for intracranial volume, total GM, total WM, and total CSF ranged between 0.996 and 0.998. ICCs for test-retest of 11 different subjects rescanned after one month segmented by one operator were > 0.98 for total GM, WM and CSF classes. ICCs for automatically obtained GM, WM and CSF volumes in lobar regions were within the range of 0.913 – 0.998. Manually delineated sub-cerebellar regions, i.e. anterior, posterior superior and inferior vermis, and hemispheres, displayed ICCs > 0.95. ICCs for the striatal structures were 0.93 for total striatum, 0.96 for caudate, 0.85 for putamen and 0.79 for nucleus accumbens.

Measures of grey matter volume were used in the statistical model. In addition, we included the ventricles, for which the volume of cerebral-spinal fluid in the central and lateral cavities was measured, the corpus callosum for which the sum of the area of white matter within the hand-traced outline of the structure on the three mid-sagittal slices (1 mm thick) was taken, and the total intracranial volume. The measures of the cerebellar lobes excluded the vermis and cerebellar tonsil regions. All measures were the sum of measurements taken from both hemispheres to eliminate issues regarding the placement of the separating plane when considering small midline structures. Clearly this precludes any analysis of lateralization or asymmetry. Our method, however, can be easily extended to cover lateralization indicies for all the morphological measurements and their interactions with diagnosis.

### Group comparisons

Group comparisons were made for all of the neuropsychological and brain morphological measures. Comparisons for a given measure were made using all subjects on which the measure had been completed. In all, eighteen neuropsychological and sixteen morphological measures were compared. Benjamini's False Discovery Rate (FDR) [30] was used to control for multiple comparisons, with the FDR set to 5%. This found a threshold for p below which the expected number of false positives was less than 5%. In determining the appropriate threshold, all of the p-values from both sets of comparisons were used.

### Morphological/cognitive relationships

#### The method

We postulated a linear regression model to express the relationship between cognition and morphology. We note that the causality implied in the model is for statistical convenience, and do not claim that it reflects biological casual effect. Dependent variables were standard neuropsychological skill tests drawn from six functional domains, four of which consisted of multiple tests as shown in table 1. A separate analysis was made for each functional domain. For those domains with multiple subtests, the dependent variable was multivariate, including the subject's scores on all of the subtests. This allowed each functional domain to be analyzed as a whole, taking into account correlations between the subtests of the given domain.

The independent variables, or covariates, consisted of the sixteen morphological measures described above. The covariate set further included the following. The total intracranial volume was included as a possible normalizing factor. Diagnosis was included as a classifier. Interaction terms between diagnosis and all of the morphological covariates were included to allow the relationships between morphometry and neuropsychology to vary between patients and controls. The covariate set also included gender and age, as they both have known associations with morphometry and also some of the neuropsychological tests [18,31]. Further, occurrence of schizophrenia is observed to be twice as common in males as in females. The data used here contained a gender bias reflective of this trend. Including gender and age in the covariate set allowed the model to adjust for these influences in the cases where they have a noticeable effect.

This model structure was analyzed to determine which of the covariates showed a strong relationship to the neuropsychological functional domain under investigation. More precisely, a search was made, for each functional domain, for covariates with good explanatory power within the context of the model. Variable search was conducted by a Bayesian decision-theoretic approach to variable selection in multivariate regressions as suggested by Brown et al. [12].

The search strategy presented in Brown et al. [12] uses a form of Stochastic Search Variable Selection (SSVS) [32]. Intuitively, SVSS can be considered a form of stepwise regression that takes many, many steps, and whose steps can both expand and contract the model. SSVS proceeds by randomly proposing to add or delete covariates to/from the model. The proposal is always accepted if it provides a better fit to the data. Proposals with a worse fit are also occasionally accepted, with odds related to how much worse the fit is. Note that the term "fit" is used loosely here. It is more proper to speak of goodness of fit, a measure which generally includes some sort of penalty on the number of parameters used as well as the sum of the squared error. SSVS tends to quickly locate high-probability regions of the model space, which it then explores. It has been shown to find better models than stepwise regression techniques when the model space is large [32-34].

SSVS is typically used in a Markov Chain Monte Carlo setting to give a picture of the posterior model probability distribution. Brown et al. [12] combine the SSVS approach with another machine learning technique called simulated annealing to instead select one high, preferably maximal, posterior probability model. In the early stages of the run the algorithm locates a region of the model space that contains highly probable models, much as in standard SSVS. Unlike traditional SSVS, however, simulated annealing progressively reduces the odds of accepting moves which slightly worsen the fit. In the later stages of the run, the algorithm performs much like a traditional stepwise selection method to select one of the better models in the previously located high-probability region. While only a total search can guarantee to find the absolute best model, this approach tends to find very good models.

SSVS uses a goodness of fit measure to decide if a proposed change to the model is accepted or not. Brown et al. [12] use a penalized error term to measure goodness of fit. Adding a covariate will always reduce the error of a model; this is counterbalanced by a penalty on the number of covariates. Penalized error has a long history in regression models, with many possible choices of penalties. Two of the most commonly used are Akaike's information criterion (AIC) [35] and the Bayesian information criterion (BIC) [36]. The choice of penalty is left to the modeler in ref. [12].

The approach is Bayesian in that prior beliefs regarding the covariance of the regression terms and the dependent variables are included in the estimate of the regression coefficients. These priors help keep the model search focused on models that are at least remotely plausible.

Brown et al. [12] tested their approach on a data set with 39 samples and 300 potential covariates. This data had previously been explored with stepwise regression. Brown et al. [12] report that the best stepwise model contained 18 covariates, and that their approach found a model with noticeably lower prediction error containing only 5 covariates [12].

#### The power of the method

A power test in classical statistics helps determine if negative findings are due to no effect, or an effect too weak to detect with the current sample size. In the context of covariate selection, the question would be if more data would cause some of the non-selected regions to be selected? Its answer depends to a large extent on the penalty term used.

The penalized error represents a balance between the squared error of the model and the number of covariates in the model. There are several methods for setting this balance. The AIC has a fixed penalty on the number of covariates regardless of the size of the sample. Adding more data increases the influence of the squared error term in determining the fit of the model. This could allow covariates with a weak effect to join the model as more data is collected.

Alternately, the penalty can adapt to the size of the data. The BIC sets the penalty as the log of the number of samples. The balance between the penalty for adding a covariate and the decrease in squared error as a result of the extra covariate is roughly constant regardless of the sample size. Borderline covariates would most likely remain on the border even with additional data.

A related question is how well does each covariate explain the data? If one considers selection based on penalized error measures, the level of penalty required to exclude the covariate is indicative of the explanatory power. A covariate which is only selected when the penalty is low has a weaker association than one that is selected even when the penalty is high. Results at different penalty levels provide an indication of the strength of the association between a selected covariate, or group of covariates, and the dependent variable. This is, of course, only a rough measure. If one is interested in directly measuring each covariate's probability of effect then a full Bayesian analysis is called for. Chipman et al. [34] provide a very nice review of the field.

### Analytical setup

Two parameter choices influence the prior probability of including a covariate. The first is the prior estimate of the variance of the regressors. Brown et al. [12] recommends setting this to a value near an estimate of the variance of the regressors of the saturated model. This setting makes it easier for the algorithm to accept new regressors by restricting proposed additions to values that are a priori reasonable.

The second parameter is the penalty term used in the goodness of fit measure. The penalty reflects a prior estimate of how many covariates belong in the model [34]. There is no obvious biological basis on which to determine an appropriate prior on the number of morphological features, which would be associated to a given neuropsychological test. Thus the results presented here were taken from multiple runs over a range of penalty levels covering commonly employed penalized error terms. Giving results at different penalty levels also provided a gauge of the strength of the association between a selected covariate, or group of covariates, and the cognitive measure. The penalty was started at 2, and increased in steps of 0.5 to 6. The value 2 corresponded to the AIC penalty, and 4 (the middle of the range) corresponded to the BIC. A penalty of 6 was, in this model setting, stronger than the common penalized error approaches, and was chosen to represent an overly strict penalty.

At each step of the search, the algorithm could propose adding a covariate, subtracting a covariate, or swapping a covariate. Each move type was given equal probability. Brown et al. [12] do not explicitly include interaction terms in their formulation, but the extension is straightforward. We allowed interactions between diagnosis and the morphological measures, but only if both main effects were in the model. When adding covariates, only eligible interactions were considered. When subtracting or swapping covariates, removal of a main effect automatically removed the associated interaction effect if one was present.

## Results

### Neuropsychology

As shown in Table 2 the patients as a group performed significantly worse than the controls in almost all test variables. Individual patients, however, were able to perform within the normal range in some tests, even achieving some of the highest scores in the RAVLT series. Along with lower mean scores, the patient group exhibited greatly increased variation compared to controls.

**Table 2:** Comparison of neuropsychological performance in controls and patients.

	**Control**			**Patient**			
		
	**Mean**	**SD**	**n**	**Mean**	**SD**	**n**	**p**
RAVLTA1	6.85	1.48	65	5.41	1.78	71	0.0059
RAVLTA2	9.74	1.73	65	7.79	2.62	71	0.0034
RAVLTA3	11.7	1.70	65	9.06	2.74	71	0.0003
RAVLTA4	12.7	1.58	65	10.1	2.75	71	0.0003
RAVLTA5	13.6	1.29	65	10.8	2.95	71	0.0003
RAVLTATOT	54.6	6.14	65	43.1	11.6	71	0.0002
RAVLTB	6.57	1.70	65	5.00	1.87	71	0.0004
RAVLTA6	12.2	2.09	65	8.80	3.36	71	0.0027
RAVLTA7	12.1	2.57	65	8.93	3.69	71	0.0054
CPT	1.13	0.61	65	0.62	0.77	70	0.0005
TMTA	23.7	7.89	65	36.1	28.8	71	0.0251
TMTB	58.4	19.1	65	102	74.9	71	0.0007
LNS	11.0	2.26	65	8.85	3.03	71	0.0008
WAIS R	50.7	11.0	65	42.68	13.6	71	0.0131
WCST CAT	3.45	1.41	65	2.45	1.65	71	0.0798
WCST total.err	15.3	8.47	65	22.8	11.7	71	0.0246
WCST pers err	7.46	4.32	65	11.8	8.27	71	0.0719
WCST pers.resp	8.06	5.32	65	13.4	10.7	71	0.0658

As a group, patients showed reduced ability in all of the tests. The difference was quite strong for all of the domains with the possible exception of the WCST. These findings are consistent with the published literature on the subject. P-values represent probability using Student's t-test (uncorrected for multiple comparisons). Applying FDR to the p-values suggests that values of p less than 0.025 are expected to have less than 5% chance of being false positives. A short description of the neuropsychological tests, including the acronyms, is given in Table 1.

The results in the RAVLT illustrate that patients with schizophrenia had difficulties learning new verbal information (RAVLTA1-5, and RAVLTATOT), to resist distraction from earlier learning (RAVLTB), to immediately recall recently learned information (RAVLTA6), and to recall verbal information after a delay (RAVLTA7). Moreover, the patients demonstrated a lower level of vigilance (CPT) and less efficient working memory (LNS), compared to the controls. Visuo-motor speed was slower (TMTA), as was the performance in the more demanding task (TMTB). The patients also made more perservative errors (WCST64perserr) and completed fewer categories (WCST64cat) than controls, demonstrating limitations in components of executive functions.

### Brain morphology

In this study, no significant volumetric differences in the major regions of the cerebral cortex were found between schizophrenic patients and controls. The caudatus and putamen both showed larger gray matter volumes in the patient group of this study sample, whereas the volume of the hippocampus was significantly smaller. Patients had smaller cerebellar vermis lobules and cerebellar tonsils gray matter volumes, but not the cerebellar lobes. The results are shown in Table 3.

**Table 3:** Comparison of brain structure volumes in controls and patients.

		**Controls**			**Patients**			
		**Mean**	**SD**	**n**	**Mean**	**SD**	**n**	**p**
1	Total intracranial volume*	1461	148	65	1500	125	71	0.10
2	Frontal cortex	250	28.5	65	257	24.5	71	0.15
3	Occipital cortex	69.7	9.07	65	71.4	8.70	71	0.27
4	Temporal cortex	149	14.9	65	149	12.9	71	0.80
5	Parietal cortex	138	18.1	65	143	13.3	71	0.088
6	Subcortical	50.7	5.82	65	52.4	4.18	71	0.047
7	Ventricles (lateral and 3rd)*	19.6	8.60	65	27.1	11.2	71	<0.0001
8	Corpus callosum*	1.57	0.24	41	1.50	0.26	63	0.20
9	Caudate	3.49	0.43	27	3.87	0.61	45	0.006
10	Putamen	5.03	0.62	27	5.57	0.64	45	0.0009
11	Hippocampus	2.68	0.39	26	2.43	0.43	37	0.022
12	Cerebellar lobes	88.8	7.58	26	91.0	7.76	38	0.25
13	Vermis (posterior inferior)	2.22	0.32	26	1.99	0.32	37	0.006
14	Vermis (posterior superior)	2.37	0.43	26	1.75	0.28	37	<0.0001
15	Vermis (anterior)	3.83	0.49	26	3.44	0.47	37	0.0027
16	Cerebellar tonsil	1.70	0.68	26	1.24	0.63	37	0.008

Affected patients show a number of gray matter reductions when compared to healthy controls. The most significant changes are enlarged ventricular volume and putamen gray matter volume, and reduced posterior superior vermis gray matter volume.

Shown are the mean and standard deviation of gray matter volumes, with the exception of the three items marked with a *. These are as follows: Total intracranial volume includes all area within the skull. The corpus callosum measure is the sum of the area of the three mid-sagittal slices. For the ventricles the volume of cerebrospinal fluid was measured. The subcortical structures (items 2–10) were measured with manual delineation, while the remaining items (11–16) were measured automatically via Talairach registration. All measures (except the corpus callosum) are in milliliters. P-values represent probability using Student's t-test uncorrected for multiple comparisons. Applying FDR to the p-values suggests that values of p less than 0.025 are expected to have less than 5% chance of being false positives.

The number of subjects varies between the comparisons, as the quantitative MRI analysis of all brain regions was not performed on all subjects. As can be seen from Table 3, more automatically measured volumes (total intracranial, cortical regions and ventricles) were available than manually drawn regions. Morphological features were compared using all available data.

### Morphological/cognitive relationships

**Figure 1:**
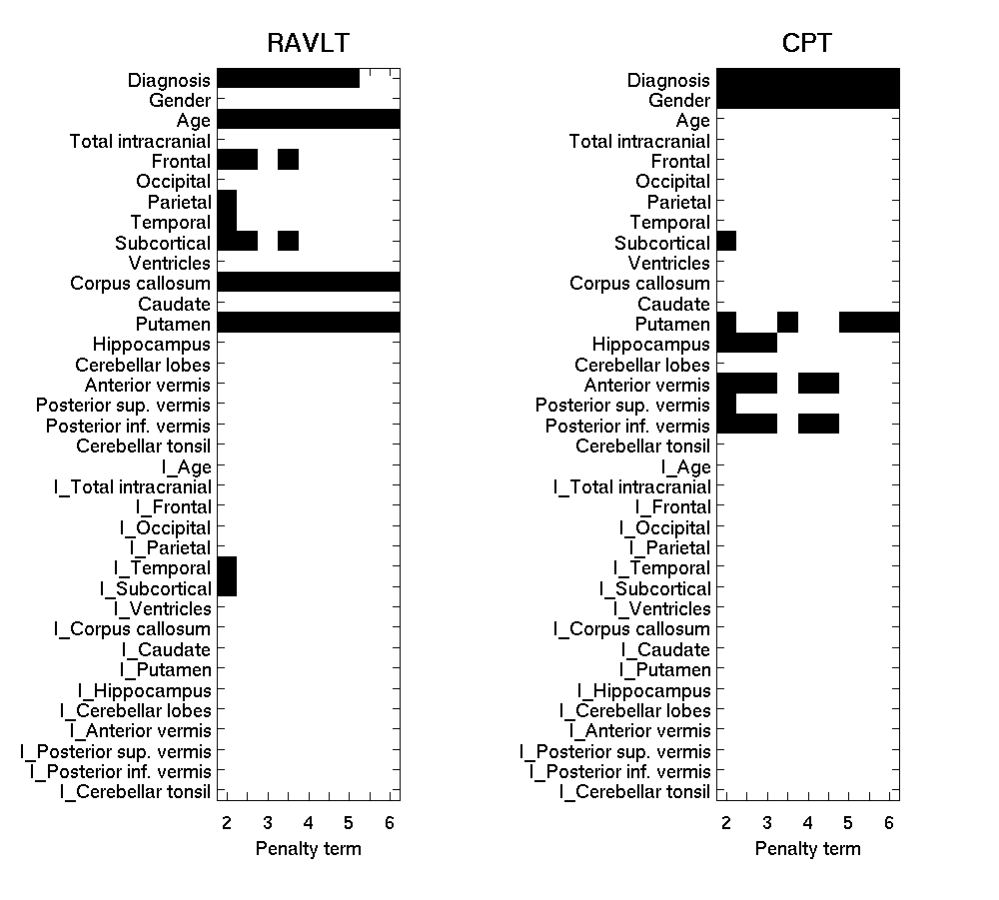
The figure shows the morphological covariates (vertical axis) selected by the algorithm over a range of goodness-of-fit penalty levels for the functional domain (Rey Auditory Verbal Learning Test) and CPT (Continuous Performing Test). The body of the plot shows, in black, which morphological features were selected for that cognitive test at the various penalty levels. The top half of the figure shows the main effects, and the bottom half (covariates with the prefix 'I') interaction with diagnosis. The penalty term is shown on the bottom axis, increasing from left to right. Covariates are often selected at several penalty levels, resulting in a black line stretching across the plot. As the penalty increases, a covariate needs more explanatory power to be selected. One should not infer that the growth in explanatory power is linear. The penalty at the left is 2, corresponding to the AIC. The middle of the graph represents a penalty of 4, the level suggested by the BIC criterion. Selections in this range represent choices that are supported by standard penalized error measures. At the right, the penalty is 6, a level stronger than called for by the common penalized error approaches. When selection switches between two covariates, as can be seen in the CPT between the putamen and the vermis (anterior and posterior inferior), the conclusion is that both groups offer nearly the same explanatory power.

**Figure 2:**
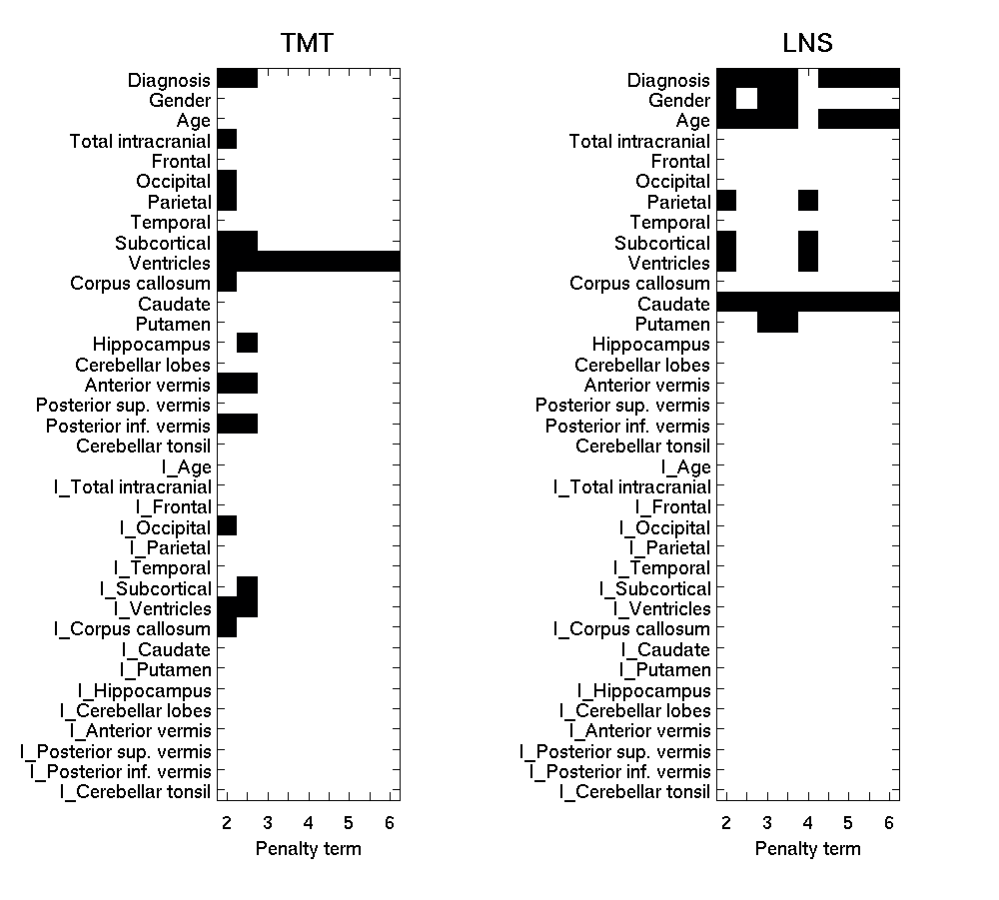
The figure shows the morphological covariates (vertical axis) selected by the algorithm over a range of goodness-of-fit penalty levels for the functional domain TMT (Trail Making Test) and LNS (Letter Number Sequencing). The body of the plot shows, in black, which morphological features were selected for that cognitive test at the various penalty levels. The top half of the figure shows the main effects, and the bottom half (covariates with the prefix 'I') interaction with diagnosis. The penalty term is shown on the bottom axis, increasing from left to right. Covariates are often selected at several penalty levels, resulting in a black line stretching across the plot. As the penalty increases, a covariate needs more explanatory power to be selected. One should not infer that the growth in explanatory power is linear. The penalty at the left is 2, corresponding to the AIC. The middle of the graph represents a penalty of 4, the level suggested by the BIC criterion. Selections in this range represent choices that are supported by standard penalized error measures. At the right, the penalty is 6, a level stronger than called for by the common penalized error approaches.

Results are presented graphically in figure 1 (RAVLT, CPT and TMT) and figure 2 (LNS, WAIS-R and WCST). The graphs show which morphological features were selected for each test over a range of penalty levels. As the penalty level increased (moving from left to right in the figure), a covariate needed more explanatory power to be selected, though the growth is not linear. Some covariates were selected for all or nearly all penalty levels. These had the strongest association with the outcome in this data. For others, selection shifted between two covariates, or groups of covariates, as the penalty increased. This indicated that the two options had nearly equal association with the outcome, though the association was not as strong as for covariates which are selected for (nearly) all of the penalty levels. We interpret the figures as follows.

Diagnostic status was selected as a covariate for almost every test. The relationship was strong for the RAVLT, CPT, and LNS test, much weaker for the TMT and WCST, and almost negligible for the WAIS-R. These last three tests, however, all strongly selected ventricular volume, which is indicative of diagnosis. Interaction terms were rarely selected for any of the cognitive tests indicating that few of the relationships were different in patients and controls. Exceptions will be noted in the presentation of findings for the individual tests.

Gender was associated with the CPT test and to a lesser degree with the LNS. Age showed some importance for the RAVLT, LNS, and WCST. As these are not morphological features and the findings are in line with the clinical literature [18,31], little more will be said about them.

Verbal learning (RAVLT) was strongly associated with the corpus callosum measure and with putamen volume. The total subcortical gray showed a weaker, though still existent relationship, as did the frontal volumes. There appeared to be a rather weak difference in the relationship of subcortical volume to performance between the two classes, as indicated by the selection of the interaction term at the lowest penalty level.

Vigilance (CPT) scores were associated with either putamen volumes or both the anterior and posterior vermis together. Since for penalties greater than 3 selection alternated between these two options, it appears that both have approximately the same explanatory power. Selection of putamen instead of the vermis regions for the highest penalties is likely due to the more parsimonious nature of this model; it has one fewer covariate.

Visuo-motor speed (TMT) showed a strong association to ventricular volume and little else. There was weaker evidence for an association with the anterior and the posterior inferior vermis, and for an interaction effect for the ventricles. No interaction was observed for the vermis regions.

Working memory (LNS) showed a strong association with caudate volume. Selection at low and medium penalties varied between the group of (diagnosis, gender and putamen) and (parietal, subcortical, and ventricles). These two covariate groups, then, had approximately the same level of relationship with the outcomes.

Vocabulary (WAIS-R) test results were linked with ventricular volume. A very weak association with diagnosis and vermis volumes (all regions) was also observed.

Executive function (WCST) showed a strong relationship to the anterior vermis. Diagnosis was only selected at low penalty levels, while the ventricles were selected at all penalty levels. The parietal and temporal lobe volumes both had an impact on test scores, with the temporal having the stronger relationship. An interaction effect was seen for the ventricles, though its strength was hard to judge as the exclusion of diagnosis at all but the lowest penalty levels automatically removed interaction from consideration. There was weak evidence for the putamen, and the relationship differed between patients and controls as indicated by the inclusion of the interaction term.

## Discussion

That patients with schizophrenia have reduced cognitive performance has been clearly demonstrated in a multitude of other works [2]. Preliminary analysis of the present data [37] also showed cognitive impairments in patients with schizophrenia in comparison to different groups of genetically related and unrelated subjects. The subject group used in this study shows no deviation from the established literature on neuropsychological testing.

It is also well known that the brain morphology is abnormal in schizophrenia. This has been demonstrated many times [3-5], and in the subject group used in this current work [7,8,26,27,38,39]. The findings presented here are generally in line with these earlier studies with one important exception. The data used in this work showed no significant differences in cortical lobe volumes, though deficiencies are commonly reported elsewhere. This could be because the group comparison was made on raw (non-normalized) data.

The first theoretical framework mentioned in the introduction is concerned with volumetric and thickness deficits in brain cortical matter in schizophrenia. The method used in this work found that frontal lobe gray matter volumes were associated with verbal learning, parietal lobe gray matter volumes were associated with working memory, and parietal and temporal lobe gray matter volumes were associated with executive function. Only for the executive learning tasks, however, was the evidence for involvement of large cortical gray-matter regions present at the highest penalty levels. This can be contrasted with findings for the putamen, caudate, and vermis volumes, which, when associated with a given neuropsychological measure, tended to be associated even at high penalty levels. That the associations of cortical regions to verbal learning and working memory were not as strong as those of the subcortical and cerebellar regions, coupled with the lack of association between the cerebellar regions and the remaining four neuropsychological domains suggests that gray matter variation in the cerebral cortex has less impact on cognitive function than variation in non-cortical structures, at least in the material studied here. This provides evidence favoring the importance of connectivity in explaining neuropsychological deficiencies in schizophrenia.

The findings could alternately reflect generality in our measures. The Talairach boxes contained large sections of the brain, whereas functional abnormalities could be localized to smaller regions. A follow-up analysis of our subjects using fine-grained cortical thickness measures is being planned. An alternate possibility is that volumetric changes in different brain regions give only one dimension of possible abnormalities in brain function. Functional abnormalities in the human brain may not always be directly related to regional volumes, but rather to changes at the ultrastructural level, in connectivity, or in neural transmission. These changes will not show up in a morphological study like this, and may be the reason for the absence of association.

We note that the striatal structures were strongly associated with verbal learning, vigilance, and working memory, and weakly associated with executive function. The suggestion is that neural circuitry involving this region plays an important role in cognition, a theoretical framework that is gathering increasing support [40]. For example, findings reported by Hokama et al. [41] show that the increased striatal volumes, and especially of the caudate, were associated with poorer neuropsychological test performance (finger tapping and Hebb's Recurring Digits). Other studies of disturbances in cortical circuits in schizophrenia have placed special focus to the circuits involving frontal cortex, thalamus, and cerebellum [1,42]. It is interesting to note the central role the striatum plays in this network. Diverse areas of the cerebral cortex converge on the striatum before being projected to the region of the frontal cortex that contributes to the striatal input, and some of the connectivity between the thalamus and frontal cortex routes through the striatum [40].

One should consider if the findings in the caudate and putamen reflect a true functional relationship or merely a side effect of neuroleptic usage in the patient group. An increase in putamen volumes has been shown after antipsychotic treatment, although these enlargements are less prominent in patients treated with atypical antipsychotics [3] and not found in all data sets [43]. Note that interactions between diagnosis and caudate or putamen volume were never associated with neuropsychological performance, with the exception of weak findings regarding the caudate and executive function. This indicates that the relationship of volume to cognition is similar in the patient and control groups for these structures, evidence against a medication-related effect. An alternate explanation also exists. Lifetime neuroleptic exposure is related to age and diagnosis, both of which were also associated for all of the neuropsychological tests associated with the either the caudate or putamen (with the exception of vigilance, which showed association to diagnosis but not age). The lack of association could be because neuroleptic related effects were substantially accounted for via these measures. A study on a group of neuroleptic-naive subjects, or subjects whose treatment has been primarily atypical medications, would be desirable to understand why striatal structures showed such strong association to the neuropsychological tests.

Neither current nor lifetime neuroleptic exposure were considered in the analysis. It is well known from many studies that the doses of antipsychotic drugs used do not induce attention deficits in patients with schizophrenia [44]. These and other deficits in attentional, cognitive and executive functions are core features of the disorder. The effects of the medication on attention may rather be positive, according to recent reviews [44]. Neurocognitive deficits are, further, not related to symptom intensity, especially not in groups of stable, chronic patients such as the subjects in this study. Exceptions may be noticed in an acute episode, but this was not the case in this study.

The selection of the corpus callosum in RAVLT indicates that cross-hemispheric communication is important for verbal learning. The corpus callosum was not found to be significantly smaller in patients in our data (see table 3), but differences in structure have been observed. Specifically, the posterior region showed a lower fractional anisotropy [39] using diffusion tensor imaging of the subjects in our sample.

The findings of the current study support earlier findings on involvement of cerebellar structures in cognitive brain function. Considerable evidence demonstrates that cerebellar structures are involved in diverse cognitive functions, including working memory, motor skill learning, explicit memory, and language [45]. Schmahmann [45] theorizes a universal cerebellar transform, which facilitates automatic modulation of behavior around a homeostatic baseline, where the behavior being modulated is determined by the specificity of anatomic subcircuits within the cerebrocerebellar system. Functions of the cerebellum have been described as not only including coordination of balance, gait, extremity and eye movements, but also dysmetria of thought, and syndromes of cognition and affect [45]. Cognitive and affective functions were postulated to have their topographical representation in the neocerebellum [45]. The neocerebellum includes vermian lobes IV-VII, their hemispheric extensions, and the ventrolateral portion of the dentate nucleus. Of the cerebellar substructures, it has been found to show the greatest increments in size with evolution. It is extensively connected with areas of the brain crucial for cognition such as the dorsolateral prefrontal cortex [46]. In the study by MacLullich et al. [46], vermis areas VI – VII were found to correlate with four out of eight test of cognitive ability in elderly healthy men, while areas of the other parts of the vermis did not correlate with cognitive ability. The posterior superior vermis of this study correspond to vermis areas VI – VII in the work of MacLullich et al. [46].

Relationships between cerebellar hypoplasia of vermis lobuli VI-VII (the posterior superior vermis), frontal lobe cognitive deficits, and visually guided eye-hand and eye movements have been previously demonstrated [47]. Our findings of an association between visuo-motor speed and vermis volumes offers additional support to the importance of this region in tasks involving visual coordination, though our findings were in the anterior and posterior inferior regions. The association between vermis gray matter volume and both vigilance and executive ability could reflect the visual components of these tasks.

In a positron emission study from Dr. Andreasen's research group, it was demonstrated that patients with schizophrenia had decreased flow in the anterior vermis during recall of both well-learned and novel word lists [48], though the authors' conclusions regarding the cognitive processes involved is open to debate [49]. In our data, no relationship was found between verbal learning and any of the vermis volumes. However, our work only considered gray matter volumes. The decreased blood flow noticed by Dr. Andreasen's group could perhaps be related to white matter abnormalities. Recent work on diffusion tensor images has shown abnormalities in related cerebellar regions in schizophrenia [50].

The selection of only the ventricles for the WAIS-R (vocabulary) was quite interesting. WAIS-R is a measure of pre-morbid neuropsychological state, as much of vocabulary is learned before the typical age of onset of schizophrenic symptoms. With reservation for the limitations of the measure, the WAIS-R tests scores seen here suggest that the decrease in cognitive ability in patients began before the onset of psychotic symptoms. Ventricular volume is strongly correlated with diagnosis in our data. That the ventricles were selected instead of diagnosis suggests that brain morphological abnormalities related to diagnosis are more closely correlated to WAIS-R scores than diagnostic outcome alone, in our data. If the WAIS-R scores reflect a pre-symptotic deficit, then the implication is that the cause of this deficit is more closely linked to morphological abnormalities related to diagnosis than to diagnosis itself.

Interaction effects were only rarely associated with any of the neuropsychological tests. This indicates that the associations found are similar in both patients and controls. Had interactions shown a strong association to neuropsychology, this would provide evidence that the nature of the morphometric/neuropsychological relationship was altered in schizophrenia. Little such evidence was found, with two slight exceptions. These are with visuo-motor speed and executive function. For both of these tasks, an interaction effect for the ventricles was noted at low penalty levels. At medium and higher penalties, however, the association of diagnosis with the test outcome was no longer observed. Apparently, diagnosis and the interaction between diagnosis and ventricular volume showed only mild relationship to variation in neuropsychological performance

### Strengths and weaknesses

A major strength of this study was that it reaches across theoretical boundaries. All of the morphological features included in the analysis have support in the literature, but this support comes from different theoretical frameworks. This study has been able to conduct an investigation that reaches across these frameworks, allowing for interrelations between the terms, and alterations in the relationships in the patient group. Exploring across and outside of established theoretical frameworks is necessary in order to discover new aspects of the etiology of schizophrenia and to reveal new associations among variables. If further progress is to be made in unraveling the etiology of schizophrenia, in the service of providing etiologically oriented treatment and prevention, then research needs to be conducted with an approach covering the whole spectrum of variables of interest.

Of course such analysis is aided by high quality data. The subjects in this study were drawn from a (by international standards) homogeneous population that was carefully investigated and clinically characterized by patient interviews, case notes from the medical records, physical examination, and neuropsychological testing. Cognitive testing was carried out by trained professionals in a supervised and standardized manner. The same calibrated MR instrument was used for all investigations without upgrading or other changes that were uncontrolled for during the sample collection time. Brain morphological measures were obtained using methods that have shown high reliability and validity in several scientific studies.

Advanced statistical analysis has provided benefit in analyzing other aspects of cognitive dysfunction [51]. They have also proved useful in treatment evaluation, for example in an investigation of alternative haloperidol dosage regimes in schizophrenia [52] and the safety and effectiveness of olanzapine [53]. Their clinical benefit has been demonstrated by Sawamura et al. [54]. Data mining approaches have found interesting results in our data as well [55].

Tests were made for associations within each functional domain by exploring a linear multivariate response model. An alternate approach would have been to use the Seeming Unrelated Regressions (SUR) model [56]. SUR is a variant of least-squares model fitting, which allows the covariance in the errors to influence the selection of the regression coefficients. It has proved very popular in econometrics. It seems to offer the most benefit in non-linear models. Tests on linear models such as used in our investigation show it to have little additional effect [57]. Preliminary tests on our own material, based on a method suggested by Holmes et al. (1999) [58] also show it to yield only marginal reductions in model error. Non-linear models, however, are also quite appealing for analyzing morphological/cognitive relationships. A SUR analysis of such a model could prove fruitful.

We elected to use penalized error to measure goodness of fit, and presented results over a range of penalty terms. The SSVS approach, however, allows any objective goodness of fit measure to be used. The AIC and the BIC, two of the penalized error measures included in our range, are approximations to the log-evidence or log-marginal likelihood of the model. It would have been possible to replace the AIC with the variational free energy.

Our model only included interaction terms when main effects were present. This was, strictly speaking, not necessary. Many factorial designs (a crossover study, for example) can result in significant interaction terms without a significant main effect.

While evidence for several morphological covariates to cognitive performance that differed in schizophrenia were found, their causes were unexplored. Are these developmental or degenerative effects? It would be of interest to test these relationships against such variables as disease duration or age of onset. Also, given the effect of neuroleptic exposure on certain volumes, it would be of interest to test if the disease-related differences are associated with medication.

A perhaps more serious weakness was the (necessary) assumption of the model that gray matter volume alone explains cognitive performance. While some connection must exist, gray matter volumes are certainly not the only factor in cognition. White matter is one obvious alternative. Our model, then, was incomplete. While we can say that our tests indicated a connection between certain morphological features and a type of cognitive skill, we cannot be certain that this connection would have the same level of importance if these other unspecified quantities had been included.

## Conclusion

This study was able to test strength of relationships between brain morphology and neuropsychological performance and their possible alterations in schizophrenia. Several interesting results were found, which provide evidence in the ongoing debate surrounding morphological relations to cognition. Further studies are warranted to determine the underlying mechanisms in schizophrenia of these observations.

## Competing interests

The author(s) declare that they have no competing interests.

## Authors' contributions

GL and HH drafted the manuscript and were responsible for its design and co-ordination. HN designed and performed the neuropsychological investigations. IA designed and performed the MRI investigations. GL and SA designed and performed the statistical analyses. EGJ designed and performed the clinical investigations. GCS conceived of the study and participated in its design and co-ordination. All authors read and participated in the revision the manuscript and have also approved the final manuscript.

**Figure 3:**
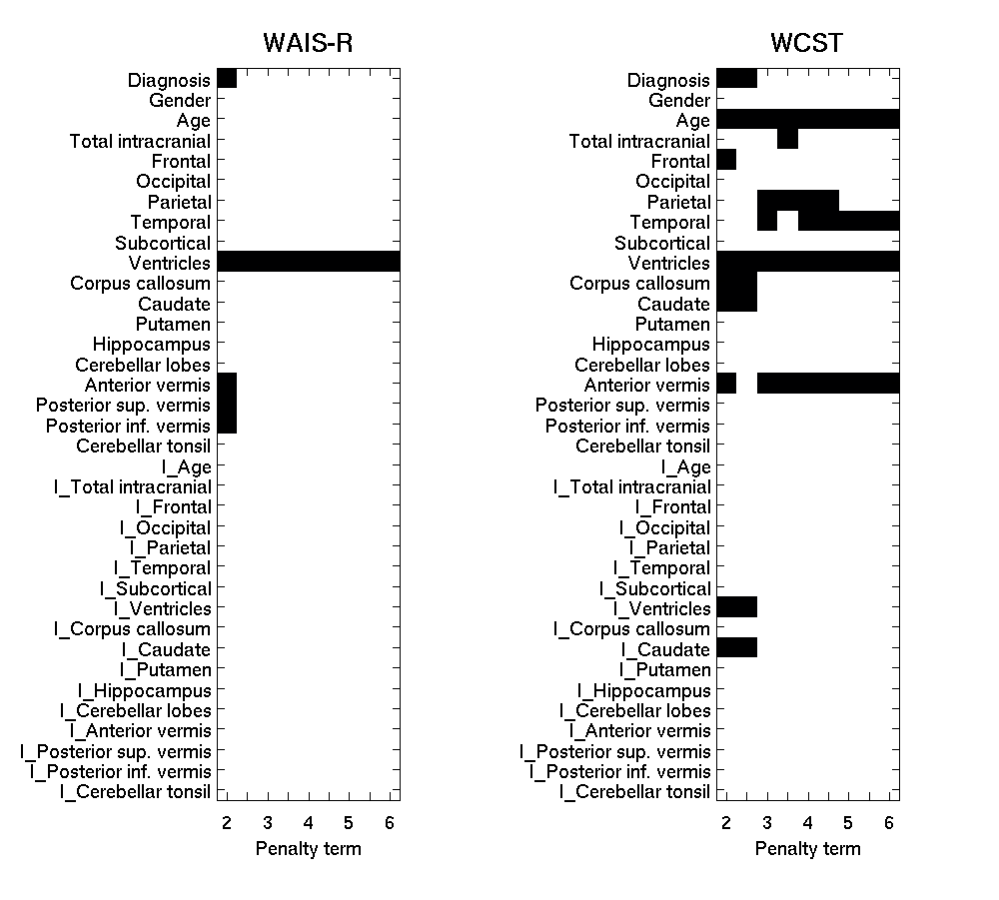
The figure shows the morphological covariates (vertical axis) selected by the algorithm over a range of goodness-of-fit penalty levels for the functional domain WAIS-R (subtest from Wechsler Adult Intelligence Scale) and WCST (Wisconsin Card Sorting Test). The body of the plot shows, in black, which morphological features were selected for that cognitive test at the various penalty levels. The top half of the figure shows the main effects, and the bottom half (covariates with the prefix 'I') interaction with diagnosis. The penalty term is shown on the bottom axis, increasing from left to right. Covariates are often selected at several penalty levels, resulting in a black line stretching across the plot. As the penalty increases, a covariate needs more explanatory power to be selected. One should not infer that the growth in explanatory power is linear. The penalty at the left is 2, corresponding to the AIC. The middle of the graph represents a penalty of 4, the level suggested by the BIC criterion. Selections in this range represent choices that are supported by standard penalized error measures. At the right, the penalty is 6, a level stronger than called for by the common penalized error approaches.

## Pre-publication history

The pre-publication history for this paper can be accessed here:


